# Modulatory effects of orexin and dynorphin on stress-related alcohol seeking and relapse: pivotal role of the posterior paraventricular nucleus of the thalamus

**DOI:** 10.3389/fphar.2026.1729040

**Published:** 2026-01-26

**Authors:** Gabriel Holguin, Rémi Martin-Fardon

**Affiliations:** Department of Translational Medicine, The Scripps Research Institute, La Jolla, CA, United States

**Keywords:** addiction, alcohol reinstatement, dynorphin, kappa receptor, orexin, orexin receptor, paraventricular nucleus of hypothalamus, stress dysregulation

## Abstract

Alcohol use disorder (AUD) remains a significant problem in the United States, resulting in over 178,000 alcohol-related deaths annually. A central problem in treating AUD is the high rate of relapse to alcohol use even after protracted periods of abstinence. Stress is a major contributor to the chronic relapsing and compulsive nature of AUD, and it alters neurocircuitry mediating craving and drug seeking. Chronic alcohol use dysregulates the neuropeptides orexin (OX)/hypocretin and dynorphin (DYN), which contribute to alcohol seeking and relapse. OX neurons originate exclusively in the hypothalamus and co-express DYN. Although OX and DYN are localized in the same synaptic vesicles and co-released when the hypothalamus is stimulated, they play opposing roles in reward, motivation, and substance use. OX, via OX receptor (OXR) signaling, promotes reward-seeking behavior, whereas DYN, acting through κ-opioid receptors (KOPs), increases depressive-like states and plays a key role in mediating aversive effects of stress. OX neurons densely innervate the paraventricular nucleus of the thalamus (PVT), a brain region that is involved in the regulation of reward function, stress, anxiety, and drug-directed behavior. In individuals with AUD, chronic alcohol use damages the thalamus, resulting in volume reductions and cognitive deficits. Therefore, lasting changes in PVT OX/DYN transmission and their interaction following chronic alcohol use may underlie stress-induced alcohol craving and relapse. Although their opposing roles in the PVT are established, implications of their interaction, particularly under conditions of stress, are limited in the context of alcohol use and reinstatement. This review synthesizes evidence from preclinical evidence and complementary clinical observations that implicate the co-transmission of OX and DYN in the PVT, with an emphasis on the posterior PVT (pPVT), which receives the most OX afferents, during the stress-induced reinstatement of alcohol seeking. We also discuss the potential of targeting OXRs and KOPs pharmacologically to reduce stress-induced alcohol craving and reinstatement. This review will help disentangle individual vs. interactive contributions of OX and DYN, and elucidate how their modulation within stress- and reward-related circuits may reveal novel insights for preventing relapse in individuals with AUD.

## 1 Overview

This review outlines individual and interactive effects of the neuropeptides orexin (OX; also called hypocretin) and dynorphin (DYN) and how their stress-modulated activity contributes to the development of alcohol use disorder (AUD) and relapse. We begin by discussing physiological properties of the OX and DYN systems and their relationship with stress and reward in the context of alcohol-related behaviors. Although OX neurons originate in the hypothalamus (HYP), this review emphasizes individual and interactive roles of OX and DYN in a major projection site, the paraventricular nucleus of the thalamus (PVT). We highlight opposing functions of OX and DYN and review emerging evidence that ties their alcohol-induced imbalance to the heightened risk of future chronic alcohol consumption and reinstatement. This review also underscores how stress dysregulates the OX and DYN systems and how this dysregulation, in combination with chronic alcohol use, further exacerbates alcohol-seeking behavior. Finally, we discuss the potential of targeting these systems pharmacologically, including the repurposing of dual OX receptor antagonists (DORAs), to reduce craving and normalize sleep disturbances, another significant predictor of stress-related alcohol relapse. This review synthesizes evidence from preclinical and clinical evidence implicating OXR and KOP signaling in AUD, with a focus on the PVT and posterior PVT (pPVT) as a stress- and alcohol-sensitive node that modulates stress-related alcohol seeking and reinstatement.

## 2 Background

AUD remains a major public health concern in the United States, accounting for over 178,000 alcohol-related deaths annually. The 2023 National Survey on Drug Use and Health indicated that 16.3 million adults aged 18 and older (6.3% of this age group) reported heavy alcohol use in the past month (Substance Abuse and Mental Health Services Administration, 2023a; Substance Abuse and Mental Health Services Administration, 2023b; Substance Abuse and Mental Health Services Administration, 2023c), and that 28.1 million adults 18+ years old (10.9% of this age group) had AUD in the past year (Substance Abuse and Mental Health Services Administration, 2023a). A critical problem in treating AUD is the high rate of relapse following alcohol use, even after protracted periods of abstinence, making arelapse prevention a central focus of medication development efforts (Dejong, 1994; O'Brien and Thomas Mclellan, 1996).

Stress is a major contributor to the chronic relapsing and compulsive nature of alcohol addiction, and it is a powerful predictor of AUD and relapse in human and rodent models (Breese et al., 2005; Nieto et al., 2021; Sinha, 2007; Weera, 2019). Although the lasting effects of stress on alcohol use are well established, the complex interactions among neuropeptide systems have yet to be fully understood. Importantly, dysregulation of the OX and DYN systems in the PVT has emerged as a critical but underexplored mechanism that links stress to alcohol-seeking behavior and relapse.

## 3 Converging neurocircuitry of stress and alcohol use

The neurocircuitry that drives craving and drug seeking includes the medial prefrontal cortex (mPFC), basolateral amygdala (BLA), central nucleus of the amygdala (CeA), bed nucleus of the stria terminalis (BNST), ventral tegmental area (VTA), nucleus accumbens (NAc), hippocampus, and dorsal striatum (Everitt and Robbins, 2016; George and Koob, 2010; Koob and Volkow, 2010), with recent evidence also implicating the PVT as a critical node in this network (Kirouac, 2015; Millan et al., 2017). Stress disrupts many of these regions, particularly the mPFC, amygdala, BNST, and NAc (Arnsten, 2009; Avery et al., 2016; Flores-Ramirez et al., 2024; Koob and Schulkin, 2019; Muschamp and Carlezon, 2013).

Another key region that has overlapping modulation through stress and alcohol use is the PVT, an underexplored structure that is emerging as a hub in stress- and addiction-related circuits, modulating responses to both acute and chronic stress (Bhatnagar et al., 2003; Bhatnagar et al., 2002; Hsu et al., 2014; Nedelescu et al., 2025; Radley and Sawchenko, 2015; Rowson and Pleil, 2021). The PVT regulates arousal, wakefulness, attentional processing, and reward value (Bentivoglio et al., 1991; Groenewegen and Berendse, 1994; Kelley et al., 2005; Van Der Werf et al., 2002). A collection of studies also supports the PVT’s integrative role in alcohol-seeking behavior by linking stress and reward circuits, such that when stress dysregulates its activity, the reinstatement of alcohol-seeking behavior vulnerability increases. Notably, literature that highlights the OX and DYN neuropeptide systems, which heavily innervate the PVT and exert opposing excitatory and inhibitory influences on substance-seeking behavior, may further explain how stress and alcohol converge on this region to drive alcohol-seeking behavior (Anderson et al., 2018; Illenberger et al., 2024; Matzeu and Martin-Fardon, 2020a).

## 4 Orexin

### 4.1 The OX system regulates stress, reward-motivated behaviors, and addiction

**FIGURE 1:**
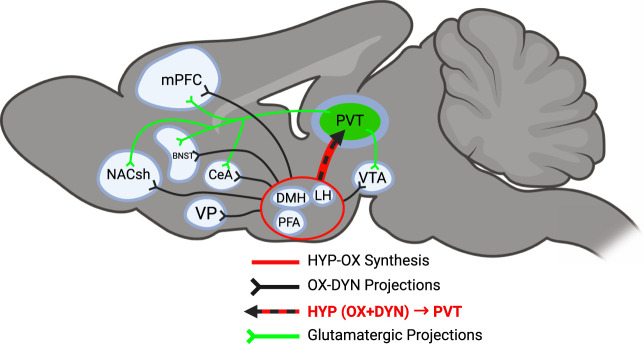
Schematic diagram of OX-DYN projections from hypothalamic OX-producing regions (red circle) to the PVT. Black lines from hypothalamic regions represent all OX-DYN projections. The red + black-dotted arrow denotes the OX-DYN projection from hypothalamic OX regions to the PVT where OX and DYN are co-released. Green lines represent glutamatergic projections from the PVT.

OX is an excitatory neuropeptide that exists in two forms, OX-A (hypocretin-1) and OX-B (hypocretin-2) (De Lecea et al., 1998; Sakurai et al., 1998b). Both are derived from the proteolytic cleavage of the common precursor prepro-OX (De Lecea et al., 1998; Gatfield et al., 2010; Sakurai et al., 1998a; Tsujino and Sakurai, 2009). Two OX receptors have been identified: OX1R and OX2R. OX-A has stronger affinity for OX1Rs and OX2Rs relative to OX-B (Ammoun et al., 2003; Sakurai et al., 1998a; Scammell and Winrow, 2011). OX is produced only in the HYP, a region that is critically involved in arousal and motivated behavior, comprising the lateral HYP (LH), dorsomedial HYP (DMH), and perifornical area (PFA), and regulates a range of physiological and behavioral functions, including arousal, sleep/wakefulness, feeding, and energy metabolism (Edwards et al., 1999; Haynes et al., 2002; Haynes et al., 2000; Sakurai et al., 1998a; Sutcliffe and De Lecea, 2002; Taheri et al., 2002; Teske et al., 2010; Willie et al., 2001). Although OX neurons originate in the HYP, they project widely throughout the PVT and other brain regions, which contributes to a multitude of physiological functions, including the mediation of motivated behavior and reward processing (De Lecea et al., 1998; Marcus et al., 2001). OX synthesis and projection sites are depicted in Figure 1. In addition to regulating arousal and motivated behavior, OXR signaling is strongly influenced by stress and contributes to stress responsivity across multiple brain regions, including the PVT, suggesting an important role in stress-related alcohol behaviors (Grafe and Bhatnagar, 2018; Heydendael et al., 2011; Kim et al., 2023).

OX neurons project to the whole PVT, with the pPVT receiving the greatest density of OX afferent terminals (Kirouac et al., 2005), and regions that participate in the regulation of drug seeking and stress response, including the NAc shell (NAcSh), ventral pallidum (VP), VTA, CeA, BNST, and mPFC (Baldo et al., 2003; Peyron et al., 1998). OX transmission influences neurobehavioral and motivational effects of drugs of abuse (Bonci and Borgland, 2009; Borgland et al., 2006; Thompson and Borgland, 2011). OX neurons become activated by food-, morphine-, cocaine-, and alcohol-related stimuli in the absence of the substance itself (Dayas et al., 2008; Harris et al., 2005; Jupp et al., 2011a; Martin-Fardon et al., 2010; Martin-Fardon and Weiss, 2017).

Greater LH OX neuron activation was associated with more intense alcohol-seeking behavior in rats during relapse-like responding for alcoholic beer (Hamlin et al., 2007; Millan et al., 2010). Similarly, OX1R blockade decreased alcohol self-administration (Lawrence et al., 2006) and the cue- and stress-induced reinstatement of alcohol seeking in rats (Jupp et al., 2011a; Lawrence et al., 2006; Richards et al., 2008; Smith et al., 2010). This suggests that OX neurons encode conditioned associations between environmental cues and reward availability, positioning them as key drivers of relapse (James et al., 2016; Mahler et al., 2012).

### 4.2 Alcohol disrupts stress-regulatory hormones and the OX system

Literature illustrates stress-inducing effects of alcohol exposure in both voluntary (Richardson et al., 2008) and forced (Rivier, 1993; Rivier et al., 1984) alcohol administration paradigms. Willey et al. (2012) reported that intraperitoneal (i.p.) alcohol administration (0.5–1.5 g/kg) dose-dependently increased plasma corticosterone levels in male and female Sprague-Dawley rats of different ages (Willey et al., 2012). This indicates activation of the hypothalamic-pituitary-adrenal (HPA) axis, a central component of the stress response that is consistent with earlier findings (Ellis, 1966; Thiagarajan et al., 1989). Increases in adrenocorticotropic hormone (ACTH) that were positively related to blood alcohol levels were also observed in response to numerous modes of alcohol delivery, including acute i.p. and intragastric administration (Ogilvie et al., 1997). Long-term disruptions of other stress peptides and pathways, particularly OX neurocircuitry that is involved in regulating stress and arousal, may facilitate chronic alcohol use and dependence.

Several reports indicate that alcohol consumption promotes OX transmission through increases in *Hcrtr* mRNA expression (Barson et al., 2015) and *Hcrt* mRNA expression (Lawrence et al., 2006; Morganstern et al., 2010) in rats and increases in OX peptides in zebrafish (Sterling et al., 2015). Following chronic voluntary alcohol consumption in male inbred alcohol-preferring (iP) rats, *Hcrt* mRNA expression was upregulated in the LH, suggesting the enhancement of OX transmission in this key arousal and motivation region (Lawrence et al., 2006). Similarly, in male Long-Evans rats, alcohol use increased *Hcrt* and *Hcrtr* mRNA expression in the HYP and anterior PVT (aPVT), respectively, indicating upregulation of the OX system during alcohol intake (Barson et al., 2015). This effect was corroborated in Wistar rats (males and females), such that intermittent alcohol vapor-induced dependence increased hypothalamic *Hcrt* mRNA expression and *Hcrtr1/2* mRNA expression at acute (i.e., 8 h) abstinence (Matzeu and Martin-Fardon, 2020a). Conversely, alcohol dependence that was induced by forced intragastric alcohol administration in male Sprague-Dawley rats (Braconi et al., 2010; Majchrowicz, 1975) decreased *Hcrt* mRNA expression at 12 h of abstinence (Sharma et al., 2020), indicating a model- and time-dependent effect on OX regulation and transcription.

In rats, the link between OX Fos activation and alcohol seeking has been observed in models of reinstatement or renewal that is triggered by alcohol-associated discriminative stimuli (Dayas et al., 2008; Moorman et al., 2016), alcohol-related environmental contexts (Hamlin et al., 2007; Millan et al., 2010; Moorman et al., 2016), yohimbine-induced stress (Kastman et al., 2016), and, to a lesser extent, discrete alcohol-paired cues (Moorman et al., 2016). Dayas et al. (2008) reported a significant increase of Fos expression in HYP OX neurons in male Wistar rats following the presentation of alcohol-associated contextual stimuli and an increase in Fos expression in the PVT (Dayas et al., 2008). In mice, Fos activation in OX neurons is also seen following alcohol sensitization (Macedo et al., 2013), suggesting that OX becomes more engaged after repeated exposure to alcohol itself. This parallels other findings that the magnitude of OX neuron activation positively correlates with motivational drive, such as the renewal of alcohol-seeking behavior following extinction (Hamlin et al., 2007; Millan et al., 2010; Moorman et al., 2016), highlighting the role of OX transmission in alcohol-motivated behaviors. Notably, direct causal evidence indicates that the inhibition of OX transmission blocks the reinstatement of drug- and alcohol-seeking behavior (Illenberger et al., 2023; Martin-Fardon and Weiss, 2014a; Martin-Fardon and Weiss, 2014b; Matzeu and Martin-Fardon, 2020b; Richards et al., 2008; Smith et al., 2009).

Human studies show higher plasma OX levels in early alcohol withdrawal compared with OX levels later in abstinence (Bayerlein et al., 2011; Ziółkowski et al., 2016), and these elevated levels correlate with psychological distress, particularly depression-like symptoms, during withdrawal (Von Der Goltz et al., 2011). Interestingly, Bayerlein et al. (2011) reported that higher peripheral blood lymphocyte *HCRT* mRNA levels were associated with less severe physical withdrawal symptoms in male and female patients, suggesting that OX transmission may be inversely related to withdrawal intensity in AUD (Bayerlein et al., 2011). Collectively, alcohol exposure and stress-related alcohol behaviors induce neuroplastic changes in the OX system that may contribute to the development of pathological compulsive alcohol-seeking behavior (Aston-Jones and Harris, 2004; Kalivas and O'Brien, 2008; Kelley and Berridge, 2002; Wanat et al., 2009).

### 4.3 OX promotes alcohol-seeking behavior

The OX system plays a pivotal role in regulating alcohol-motivated behavior. Several reviews have delineated its unique roles in alcohol consumption and withdrawal following dependence (Barson and Leibowitz, 2016; Brown and Lawrence, 2013; Capó et al., 2025; Lawrence, 2010; Mahler et al., 2012; Moorman, 2018; Walker and Lawrence, 2016). OX1R antagonism significantly decreased the motivation to consume alcohol in models of high vs. low alcohol-preferring rats and mice (Alcaraz-Iborra et al., 2017; Moorman et al., 2017), in rats and mice that were genetically modified to prefer alcohol or trained to binge drink (Anderson et al., 2014; Dhaher et al., 2010; Olney et al., 2015), and in alcohol-dependent mice that underwent alcohol vapor exposure (Lopez et al., 2016).

OX1R antagonists powerfully attenuate alcohol seeking and reinstatement across multiple paradigms, particularly under conditions of high motivational demand, including cue-induced responding, pharmacological stress, discriminative stimuli, and alcohol-conditioned reward (Jupp et al., 2011a; Lawrence et al., 2006; Macedo et al., 2013; Richards et al., 2008). Consistent with a state-dependent profile, these effects are most evident in operant models requiring increased effort or following alcohol dependence, whereas effects on low-level, non-pathological drinking are less consistent (Alcaraz-Iborra et al., 2017; Anderson et al., 2014; Capó et al., 2025; Dhaher et al., 2010; Lei et al., 2016; Lopez et al., 2016; Moorman et al., 2017).

### 4.4 Functional differences between OX1R and OX2R in alcohol-motivated behavior

Functional differences between OX1Rs vs. OX2Rs were suggested (Aston-Jones et al., 2010), although attributing specific alcohol-related behaviors to individual receptor subtypes remains challenging. OX1Rs are primarily implicated in alcohol-motivated behavior, particularly under conditions of high effort, stress, or reinstatement vulnerability, whereas OX2R signaling appears to contribute to alcohol consumption and conditioned reward in a region- and paradigm-specific manner (Alcaraz-Iborra et al., 2017; Anderson et al., 2014; Moorman, 2018; Olney et al., 2017; Srinivasan et al., 2012).

Selective OX2R antagonists have been shown to reduce alcohol consumption and self-administration in rodents, but effects are region- and model-specific. For example, intracerebroventricular (i.c.v.) and intra-NAc core administration of the OX2R antagonist TCSOX229 reduced alcohol self-administration in male iP rats (Brown et al., 2013), and microinjections of TCSOX229 in the aPVT decreased intermittent-access alcohol drinking in male Long-Evans rats (Barson et al., 2015). Similarly, OX2R antagonism attenuated the stress-induced reinstatement of alcohol seeking when targeted to the nucleus incertus and reduced alcohol-induced conditioned place preference and its reinstatement following systemic administration, supporting a role for OX2Rs in alcohol consumption and select conditioned reward processes in a circuit-dependent manner (Kastman et al., 2016; Shoblock et al., 2011).

Olney et al. (2017) directly compared OX1R and OX2R antagonism in the VTA and CeA of male C57BL/6J mice and found site-specific effects. The blockade of OX1Rs in the VTA reduced binge-like alcohol drinking, whereas OX2R blockade had no significant effect (Olney et al., 2017). In contrast, OX2R blockade in the aPVT reduced OX-A- and OX-B-induced increases in alcohol intake, whereas OX1R antagonism did not (Barson et al., 2015). These data illustrate the region- and paradigm-specific roles of OX1Rs and OX2Rs in targeting rewarding and motivational effects of alcohol consumption and reinstatement (Anderson et al., 2014; Brown et al., 2013; Kastman et al., 2016; Olney et al., 2017; Shoblock et al., 2011). Taken together, these findings suggest that OX1R antagonism preferentially reduces pathological or dependence-related alcohol seeking, whereas OX2R antagonism more broadly attenuates alcohol-related behaviors in a region- and task-dependent manner.

## 5 Dynorphin

### 5.1 Functional role of the KOP ligand

The DYN/KOP system is widely distributed in the central nervous system (Watson et al., 1982) and plays a key role in the development of behavioral alterations that are consistent with the “dark side” of addiction or the aversive emotional state that accompanies dependence (Bruchas et al., 2010; Carroll and Carlezon, 2013; Chavkin and Koob, 2016; Koob, 2015; Land et al., 2008). DYN, derived from the *Pdyn* gene, is processed into DYN-A and DYN-B peptides, with variants like DYN-A (1–17), DYN-A (1–8), and DYN-B (1–29). These DYN variants preferentially bind to KOPs (Chavkin et al., 1982) but also show affinity for μ-opioid (MOP) and δ-opioid (DOP) receptors (Fallon and Leslie, 1986; Schwarzer, 2009).

Unlike OX, which is synthesized primarily in the HYP, DYN is produced in a range of brain areas beyond the HYP, including mesolimbic structures that are implicated in addiction (Bali et al., 2014; Watson et al., 1982). In the human brain, *PDYN* mRNA is strongly expressed in reward-related regions, such as the dorsal and ventral striatum, NAc, amygdala, and mPFC (Hurd, 1996). DYN projections (e.g., NAc to VTA), can modulate dopamine signaling relevant to drug-motivated behavior (Shippenberg et al., 2001). *Oprk1* mRNA, which encodes the KOP, is prominent in the midline thalamic nuclei, including the PVT, and is associated with robust KOP binding (Mansour et al., 1994). This anatomical mapping positions the PVT as a critical node where KOP signaling can regulate motivational and stress-related states, supporting its therapeutic relevance (Butelman et al., 2012; Tejeda et al., 2012).

While HYP-OX neurons represent one source of DYN input to the PVT, anatomical evidence suggests that DYN-expressing neurons from additional regions, including the mPFC, insular cortex, extended amygdala, and brainstem nuclei such as the nucleus of the solitary tract, also project to the PVT, positioning this region as a convergence point for multiple DYN inputs (Hsu et al., 2014; Kirouac, 2015; Penzo and Gao, 2021). The relative contribution of these distinct DYN sources to stress-related alcohol behaviors, particularly within PVT subregions, remains largely unexplored and an important direction for future investigation.

### 5.2 DYN regulates fear and stress response

DYN/KOP expression increases in response to stress across several brain regions, including the HPA axis that modulates glucocorticoid release in animal models (Bali et al., 2015; Van’T Veer and Carlezon, 2013), humans (Ur et al., 1997), and nonhuman primates (Pascoe et al., 2008). In restraint/immobilization stress and learned helplessness paradigms, *Pdyn* mRNA expression is upregulated in the NAc, hippocampus, and HYP, which exclusively synthesizes OX (Bali et al., 2014; De Lecea et al., 1998; Lucas et al., 2011; Palkovits, 2000; Shirayama et al., 2004). In preclinical studies, KOP agonists trigger taste aversion and conditioned place aversion (Mucha and Herz, 1985), and KOP activation mediates depressive-like behavior in male Sprague-Dawley rats via increased forced swim test immobility and elevated ICSS thresholds (Carlezon et al., 2006), suggesting that KOP activation contributes to dysphoria and aversive states. Models that use KOP antagonists support this finding, showing that KOP blockade reduces anxiety- and depressive-like behavior in stressed rodents and exerts analgesic effects in models of forced swim and restraint stress (Knoll et al., 2007; McLaughlin et al., 2003; Shirayama et al., 2004; Suh et al., 2000).

KOP agonists produce aversive and dysphoric effects in humans (Leconte et al., 2022; Pfeiffer et al., 1986; Walsh et al., 2001). Patients who were given butorphanol, a mixed KOP/partial-MOP agonist, reported markedly heightened negative affect on a subjective measure of dysphoric symptoms (Schlaepfer et al., 1998), while other KOP agonists, including enadoline and ketocyclazocine, induced visual distortions, dissociation, and dysphoric states (Kumor et al., 1986; Walsh et al., 2001), further indicating a pivotal role of the DYN/KOP system in mediating dysphoria and negative affect across species, contributing to vulnerability to alcohol use and relapse.

### 5.3 Alcohol upregulates DYN, and KOP signaling blunts reward circuits

DYN/KOP expression and signaling are highly responsive to alcohol exposure and play a complex role in modulating alcohol-seeking behavior (Bloodgood et al., 2021; Butelman et al., 2012; Haun et al., 2022; Karkhanis and Al-Hasani, 2020; Karpyak et al., 2013; Wee and Koob, 2010). In paradigms of chronic alcohol consumption and withdrawal in rodents, *Pdyn* mRNA increased across several brain regions that are implicated in stress and reward, including the CeA (Zhou et al., 2013), HYP (Barson and Leibowitz, 2016; Gustafsson et al., 2007), and NAc (Przewłocka et al., 1997). During acute withdrawal following alcohol self-administration and 4 months of chronic alcohol vapor exposure, *Oprk1* mRNA expression was upregulated in the BNST in male Wistar rats (Erikson et al., 2018). Furthermore, in dependent animals, these molecular changes were accompanied by enhanced KOP signaling and functional impairments, including deficits in working memory that are reversed by KOP antagonism (Wei et al., 2022).

At the neurochemical level, alcohol exposure increases KOP sensitivity within mesolimbic reward circuits. Acute and repeated alcohol administration elevated DYN release and tissue content in the NAc, producing long-lasting adaptations that persisted into abstinence (Lindholm et al., 2000; Marinelli et al., 2006). In male macaques, chronic voluntary alcohol drinking enhanced KOP-mediated inhibition of dopamine release in the striatum, indicating increased sensitivity of KOP control over reward signaling (Siciliano et al., 2015).

Functionally, KOP agonists U50,488H and bremazocine reduced alcohol intake in male Lewis and Wistar rats (Lindholm et al., 2001; Nestby et al., 1999), and KOP antagonism increased alcohol consumption in male high alcohol-preferring Lewis rats, further underscoring the reward-dampening effects of DYN/KOP signaling. Lasting neuroadaptations of the DYN/KOP system may persist long after the termination of alcohol exposure, increasing its sensitivity to respond to future stress and in turn enhancing vulnerability to the reinstatement of alcohol-seeking behavior (Bruchas et al., 2010; Wee and Koob, 2010). Alcohol also directly altered KOP interactions with the plasma membrane lipid environment, while naltrexone counteracted these effects through both receptor- and membrane-dependent mechanisms, providing a mechanistic explanation for KOP involvement in AUD (Oasa et al., 2024).

Consistent with its role in alcohol reward, KOP activation broadly suppresses reward-related dopamine signaling across drugs of abuse. For example, the systemic administration of the KOP agonist salvinorin A elevated intracranial self-stimulation thresholds (Carlezon et al., 2006), and attenuated drug-induced dopamine release, indicating reduced reward sensitivity across substances through the suppression of dopamine transmission in the NAc and mesocortical projections to the PFC (Maisonneuve et al., 1994; Margolis et al., 2006; Shippenberg et al., 2001; Xi et al., 1998). Similarly, acute KOP activation by systemic salvinorin A and CI-977 (enadoline) administration lowered rewarding effects of intracranial self-stimulation of the medial forebrain bundle in male Wistar and Sprague-Dawley rats (Potter et al., 2011).

### 5.4 States of dependence and stress modulate DYN’s role in alcohol consumption

Consistent with evidence linking stress circuitry to alcohol dependence, the role of KOP signaling in reinforcing effects of alcohol varies as a function of stress and dependence (Koob, 2008). Models of stress-induced reinstatement show that heightened DYN/KOP activity can worsen stress and dysphoria, thereby increasing vulnerability to the reinstatement of alcohol-seeking behavior (Wee and Koob, 2010). Gillett et al. (2013) showed that systemic administration of norBNI reduced stress-induced anxiety-like behavior in male Wistar rats following chronic alcohol exposure (Gillett et al., 2013), supporting the idea that high DYN/KOP activity during withdrawal may facilitate the stress-induced reinstatement of alcohol-seeking behavior. Importantly, reviews indicate that DYN/KOP signaling exerts state-dependent effects on alcohol-related behavior by suppressing intake acutely while promoting negative affect and reinstatement vulnerability following dependence or stress (Anderson and Becker, 2017; Karkhanis and Al-Hasani, 2020; Pirino et al., 2023; Walker et al., 2012).

Acute treatment with KOP agonists generally suppresses alcohol intake, although the sustained, continuous infusion of a KOP agonist, enadoline, facilitated relapse-like drinking after abstinence in alcohol-experienced male Wistar rats (Hölter et al., 2000). Conversely, KOP antagonism preferentially attenuated withdrawal- and relapse-related drinking without suppressing baseline intake in dependent animals (Uhari-Väänänen et al., 2019; Walker and Koob, 2008), though it can increase unlimited-access alcohol intake in high-drinking male Long-Evans rats (Mitchell et al., 2005). More recently, KOP antagonism selectively reduced alcohol drinking in male and female Wistar rats with a history of alcohol dependence (Flores-Ramirez et al., 2024), highlighting the dependence-sensitive modulation of the DYN/KOP system.

Across species, KOP activation consistently dampens alcohol reward. In rodents (Logrip et al., 2009; Matsuzawa et al., 1999) and nonhuman primates (Cosgrove and Carroll, 2002), KOP agonists reduced alcohol self-administration and conditioned reward, whereas KOP antagonism can enhance reward under some conditions. Together, these findings indicate that the DYN/KOP system regulates alcohol reward and is recruited by stress to potentiate alcohol’s motivational value. Importantly, these effects vary across dependence states, stress contexts, and acute vs. sustained KOP activation, reconciling reports of both intake-suppressing and relapse-promoting outcomes.

Notably, emerging evidence indicates that KOP signaling and stress responsivity exhibit important sex-dependent differences that may influence alcohol-related behaviors. Preclinical work reports sex differences in KOP-mediated stress responses, affective behavior, and relapse vulnerability, with female rodents often showing heightened stress sensitivity and distinct behavioral responses to KOP modulation compared to males (Chartoff and Mavrikaki, 2015; Robles et al., 2014; Russell et al., 2014). These findings highlight sex as a critical biological variable in DYN/KOP function and underscore the need to delineate sex-dependent effects across dependence states and stress-related alcohol use.

## 6 OX and DYN: a push-pull relationship

### 6.1 Physiologically opposing roles of OX and DYN

The OX and DYN/KOP systems are widely distributed in the central nervous system (Watson et al., 1982) and implicated in the regulation of mood, motivation, and stress-related behaviors (Bruijnzeel, 2009; Tejeda et al., 2012), suggesting a viable therapeutic target to treat various neuropsychiatric disorders (Butelman et al., 2012; Knoll and Carlezon, 2010; Tejeda et al., 2012; Walker and Koob, 2008; Wee and Koob, 2010). Nearly all hypothalamic cells (94%) that express *Hcrt* mRNA also co-express *Pdyn* mRNA (Chou et al., 2001; Li & Van Den Pol, 2006), and the co-release and opposing effects of both peptides have been observed with electrical stimulation of the HYP (Li & Van Den Pol, 2006; Muschamp et al., 2014). However, they play opposing roles in cocaine self-administration, brain stimulation reward, impulsivity, and VTA neuron firing rate (Muschamp et al., 2014; Reeves et al., 2022; Shippenberg et al., 2001), suggesting that OX and DYN may interact in brain regions to which OX neurons project (Figure 1) and exert opposing, push-pull influences on downstream targets under specific circuit and behavioral conditions. In general, OX promotes arousal, reward seeking, and drug motivation (Lawrence et al., 2006), whereas DYN is linked to reduced reward sensitivity and enhanced stress-related aversion (Land et al., 2008; Bruchas et al., 2010).

At a cellular level, it was shown that OX depolarized target HYP neurons and increased firing, whereas DYN hyperpolarized and inhibited neuronal activity, with both opposing effects observed within the same neurons (Li & Van Den Pol, 2006). Electrophysiological and optogenetic studies also demonstrated that co-activation of OX and DYN signaling produced net-neutral effects on dopamine neuron firing, while selective blockade of OX1Rs or KOPs shifted this “balance” toward excitation or inhibition, respectively (Mohammadkhani et al., 2024; Muschamp et al., 2014). A similar effect was found in Pitx3-GFP mice, in which OXR and KOP signaling from the LH exerted opposing effects on VTA dopamine neuron firing, producing a functional counterbalance that regulates dopaminergic output (Baimel et al., 2017).

Here, the term “balance” is used conceptually to describe the relative influence of OXR- and KOP-sensitive signaling within a given circuit or physiological context. Importantly, some apparent oppositional effects attributed to DYN on OX transmission may also arise from indirect modulation via stress-related neuromodulators, including CRF (Li et al., 2010; Van’T Veer and Carlezon, 2013; Winsky-Sommerer et al., 2004), underscoring the need to distinguish other peptide interactions within the PVT. Together, the co-expression and opposing actions of OX and DYN have been central to the hypothesis that these systems are functionally antagonistic yet complementary in contributing to alcohol relapse, with effects that depend on stress, dependence, and circuit engagement (Farahbakhsh and Siciliano, 2026).

### 6.2 Functionally opposing roles of OX and DYN

When exploring the consequence of chronic alcohol on OX/DYN functional outcomes, embryonic alcohol exposure in zebrafish increased OX neurons that lacked DYN co-expression relative to controls in the anterior HYP (Yasmin et al., 2023), creating an imbalance of OX that outweighed inhibitory effects of DYN on alcohol-seeking behavior. This imbalance, marked by a weakened DYN system, may promote heightened vulnerability to circuits that underlie alcohol use and relapse.

In agreement with this developmental finding, studies in adult Wistar rats demonstrated that OX-DYN interactions within the pPVT directly regulate drug-seeking behavior at both the cellular and behavioral levels (Matzeu and Martin-Fardon, 2018). The systemic administration of the OXR1 antagonist SB334867 in adult male Wistar rats increased intracranial self-stimulation thresholds that were driven by LH stimulation in male C57BL/6J mice, indicating lower reward sensitivity. KOP blockade with norBNI alone did not alter intracranial self-stimulation thresholds, but pretreatment with norBNI blocked the SB334867-induced elevation (Muschamp et al., 2014). These findings support our push-pull model in which OXR blockade unmasks DYN-mediated reward inhibition, while KOP blockade removes this inhibitory constraint and restores reward sensitivity, a mechanism that may extend to excessive alcohol motivation and reinstatement.

## 7 Anatomically and functionally distinct roles of the aPVT and pPVT

The PVT, part of dorsal midline thalamic nuclei, plays a significant role in the regulation of arousal, wakefulness, attentional processing, and reward value (Bentivoglio et al., 1991; Groenewegen and Berendse, 1994; Kelley et al., 2005; Van Der Werf et al., 2002). Kelley et al. (2005) proposed that the PVT is a key relay that gates OX-coded reward-related communication between the HYP and both the ventral and dorsal striatum (Kelley et al., 2005). PVT subregions share many of the same inputs and outputs, but there are anatomical and functional differences in the density and strength of these projections, supporting their differential roles in regulating arousal, reward, and stress-related behaviors (Barson et al., 2020).

The entire anteroposterior PVT receives OX inputs, but the pPVT receives the densest, highlighting its stronger role in stress- and arousal-related processing (Kirouac et al., 2005). The pPVT receives greater inputs from the prelimbic cortex, IL, and posterior insular cortex, which provide information that is related to executive function, taste/sensory integration, and visceral sensation (Kirouac, 2015; Li and Kirouac, 2012). Importantly, the insula has been strongly implicated in interoceptive processing, stress sensitization, and drug craving, and its connectivity with the pPVT positions this pathway as a potential contributor to stress-driven relapse vulnerability (Bach et al., 2024; Herman, 2023). Direct evidence linking insula-pPVT signaling to alcohol relapse remains limited, highlighting an important target for future investigation.

Relative to the aPVT, the pPVT sends more projections to the ventromedial NAc shell, a region that is linked to negative affect (Dong et al., 2017). However, the aPVT receives greater inputs from the ventral subiculum and infralimbic cortex, which convey information about motivational states and arousal, respectively. The aPVT projects widely to limbic areas, with denser projections to the suprachiasmatic nucleus, which critically regulates circadian rhythm. However, projections from the pPVT are focused on the extended amygdala, particularly the BNST and CeA, key regions that are involved in fear, anxiety, and stress reactivity (Dong et al., 2017; Li and Kirouac, 2008; Moga and Moore, 1997; Vertes and Hoover, 2008).

Although these subregions have distinct projections, both the aPVT and pPVT innervate the NAc (Hamlin et al., 2009), but their target zones differ. For example, the aPVT preferentially innervates the dorsomedial shell of the NAc, which is typically linked to appetitive reward-seeking behaviors, whereas the pPVT targets the ventromedial shell, a region that is associated with aversive and avoidance behaviors (Dong et al., 2017). Although co-lateralization and functional overlap exist, these anatomical distinctions support a model in which the aPVT primarily modulates arousal, circadian rhythm, and reward-driven behaviors, whereas the pPVT specializes in processing negative affect, stress, and aversive stimuli.

Early findings by Ryabinin and Wang (1998) demonstrated that alcohol exposure activates the PVT, measured by an increase in Fos expression, laying the groundwork for functional segmentation of the aPVT vs. pPVT (Ryabinin and Wang, 1998). More recent literature shows that both the aPVT and pPVT contribute to alcohol-related behaviors but in functionally distinct ways. Pandey et al. (2019) found that alcohol-naive male Long-Evans rats that were prone to alcohol vulnerability, measured by an increase in rearing, had lower *Nts* (neurotensin) mRNA expression and peptide levels in the pPVT but not aPVT. Interestingly, intra-pPVT but not intra-aPVT Nts administration suppressed alcohol intake selectively in high-drinking rats. These findings suggest that the pPVT contributes to the vulnerability of excessive alcohol use, whereby enhancing Nts transmission may constrain drinking in high-drinking individuals (Pandey et al., 2019). Although few studies have explicitly isolated the aPVT vs. pPVT in chronic alcohol use and alcohol seeking, converging evidence indicates that the pPVT is especially recruited during chronic, high-alcohol use and dependence, and the stress-induced reinstatement of alcohol-seeking behavior (Barson et al., 2020; Dong et al., 2017; Matzeu and Martin-Fardon, 2020a; Pandey et al., 2019; Zhao et al., 2024).

## 8 Modulatory role of the pPVT in stress response

### 8.1 The pPVT regulates downstream systems that regulate stress

The pPVT exhibits heightened responsiveness to both chronic and acute stressors (Barson et al., 2020; Beck and Fibiger, 1995; Choi et al., 2019; Zhu et al., 2022). Barson et al. (2020) demonstrated preferential recruitment of the pPVT vs. the aPVT during stress-related states, with dense hypothalamic OX input (Barson et al., 2020). In male Sprague-Dawley rats, acute restraint stress following chronic intermittent cold stress selectively increased Fos expression in the pPVT relative to the aPVT (Bhatnagar and Dallman, 1998), while pPVT lesions enhanced anxiety-like behaviors, including defensive burying, after chronic restraint stress (Bhatnagar et al., 2003). Consistent with these findings, noxious mechanical stimulation (Bullitt, 1990) and food deprivation-induced stress (Timofeeva and Richard, 2001) elicited greater and earlier Fos activation in the pPVT compared with the aPVT, supporting a specialized role for the pPVT in stress-related processing.

The pPVT is increasingly seen as a hub that links signals from the HYP with stress-related brain circuits, highlighting its direct role in shaping stress responses (Hsu et al., 2014). Following chronic repeated restraint stress, the pPVT moderates plasma corticosterone responses, while leaving responses to acute stress unaffected (Jaferi et al., 2003). In parallel, glucocorticoid signaling in the pPVT was essential for HPA axis habituation to repeated or chronic stress, in which blocking glucocorticoid and mineralocorticoid receptors prevented habituation, whereas corticosterone implants enhanced it, without affecting acute stress responses (Jaferi and Bhatnagar, 2006). These data suggest that the pPVT robustly mediates stress responses after chronic or repeated stress exposure, and through its projections to the amygdala and stress-regulatory circuits (Zhao et al., 2024), may contribute to stress-driven alcohol-seeking behavior during dependence and abstinence (Barson et al., 2015; Barson et al., 2020; Hartmann and Pleil, 2021).

### 8.2 OX transmission in the pPVT regulates stress responsivity

OX inputs to the pPVT arise primarily from neurons in the HYP (Kirouac et al., 2005; Li and Kirouac, 2008), serving as a bridge between hypothalamic homeostatic systems and subsequent thalamic and forebrain nodes that modulate stress responsivity and reward processing. Using a repeated swim stress paradigm in male Sprague-Dawley rats, Heydendael et al. (2011) demonstrated that OXR signaling in the pPVT is required for stress sensitization. OX1R blockade in the pPVT during repeated swim stress, but not immediately prior to a novel restraint stressor, prevented subsequent HPA axis activation and attenuated swim stress-induced increases in HYP *Crh* mRNA expression (Heydendael et al., 2011). This indicates that OXR signaling in the pPVT during repeated stress is necessary for the brain to respond to future stressors. Blocking OXR signaling prevents this stress-sensitization process. Overall, the literature underscores a significant role of pPVT-OX transmission that influences downstream stress circuits associated with stress sensitization and stress-biased motivational states, such as the amygdala and HYP (Barson et al., 2020; Bhatnagar et al., 2002; Flores-Ramirez et al., 2025; Hartmann and Pleil, 2021; Hsu et al., 2014).

## 9 OX and DYN in the pPVT regulate alcohol-motivated behaviors, particularly those mediated by stress

Addiction research on the co-release and opposing interactions of OX and DYN has primarily focused on the mesocorticolimbic system, particularly the VTA (Borgland et al., 2006; Mieda and Sakurai, 2012; Muschamp et al., 2014; Thomas et al., 2022). Far less is known about how these systems interact in the pPVT, particularly in the context of alcohol use and relapse. The PVT regulates responses to stress and anxiety (Kirouac, 2021; Zhou and Zhu, 2019), two key contributors to the chronic relapsing nature of AUD (Higley et al., 2012), positioning OXR and KOP signaling within the pPVT as candidate substrates for stress-induced alcohol seeking.

The literature indicates that the PVT and PVT-OX transmission drives relapse-like behavior, especially under conditions of stress or cue-induced reinstatement. Hamlin et al. (2009) showed that the context-induced the reinstatement of alcoholic beer seeking in male Long-Evans rats engaged a PVT-NAcSh circuit, and that PVT lesions reduced reinstatement (Hamlin et al., 2009). Other work confirmed that inactivation of the anterior thalamus and PVT suppressed the context-induced reinstatement of alcohol seeking, supporting its role in associative relapse vulnerability (Marchant et al., 2010). Given the pPVT’s dense OX afferents (Kirouac et al., 2005; Parsons et al., 2006) and inputs from the PFC, it is well positioned to gate stress- and reward-driven alcohol seeking (Li and Kirouac, 2012).

Pivotal work has begun to uncover the relevance of OX transmission in the pPVT for alcohol- and stress-related behaviors. Matzeu and Martin-Fardon (2020a) reported that OX transmission in the pPVT is necessary for the stress-induced reinstatement of both alcohol and sweetened condensed milk seeking selectively in alcohol-dependent animals (Matzeu and Martin-Fardon, 2020a). This dependence-specific effect was accompanied by increases in *Hcrt* mRNA expression in the HYP and *Hcrtr1/2* mRNA expression in the pPVT, suggesting maladaptive recruitment of this circuit following chronic alcohol exposure and protracted abstinence. Flores-Ramirez et al. (2022) extended these findings, reporting that the oral administration of the DORA suvorexant reduced alcohol self-administration and prevented the stress-induced reinstatement of alcohol seeking only in alcohol-dependent rats (Flores-Ramirez et al., 2022), implying lasting upregulation of the OX system. This highlights how chronic alcohol use may “sensitize” OXR signaling in the pPVT, which could increase the susceptibility to stress-induced reinstatement.

Literature that highlights the roles and interactions of OX-DYN transmission in models of chronic cocaine use may extend to alcohol use and reinstatement paradigms. Matzeu et al. (2018) found that DYN suppressed OX-induced increases in glutamatergic transmission, and that intra-pPVT OX administration reinstated cocaine-seeking in a manner blocked by the co-infusion of DYN (Matzeu et al., 2018). Importantly, this effect was selective to drug-seeking behavior, suggesting that OXR/KOP signaling in the pPVT may preferentially regulate drug seeking relative to nondrug reward under some conditions. While derived from cocaine paradigms, this functionally oppositional evidence of OXR/KOP signaling in the pPVT provide a mechanistic framework, which may be extrapolated to alcohol-motivated behaviors.

The DYN/KOP system modulates reward circuits by suppressing dopamine transmission in the NAc and PFC (Maisonneuve et al., 1994; Margolis et al., 2006; Shippenberg et al., 2001; Xi et al., 1998) and by reducing reward sensitivity and opposing increases in drug-induced dopamine in the medial forebrain bundle. Given the dense projections from the pPVT to BNST and CeA and strong innervation by HYP OX/DYN neurons (Hsu et al., 2014; Li and Kirouac, 2008; Penzo and Gao, 2021), pPVT KOP blockade may dampen alcohol-motivated behavior during negative affect states of withdrawal and reinstatement. Indeed, KOP agonists generally reduce alcohol intake (Lindholm et al., 2001; Nestby et al., 1999), whereas KOP antagonists selectively attenuate dependence-related drinking and stress-induced reinstatement (Flores-Ramirez et al., 2024; Koob, 2008; Wee and Koob, 2010). Heightened DYN activity is linked to stress-induced anxiety post-dependence (Gillett et al., 2013), and DYN decreases neuronal excitability in the pPVT, suggesting that this region may serve as a critical hub where stress-recruited DYN/KOP signaling promotes relapse (Chen et al., 2015).

**FIGURE 2:**
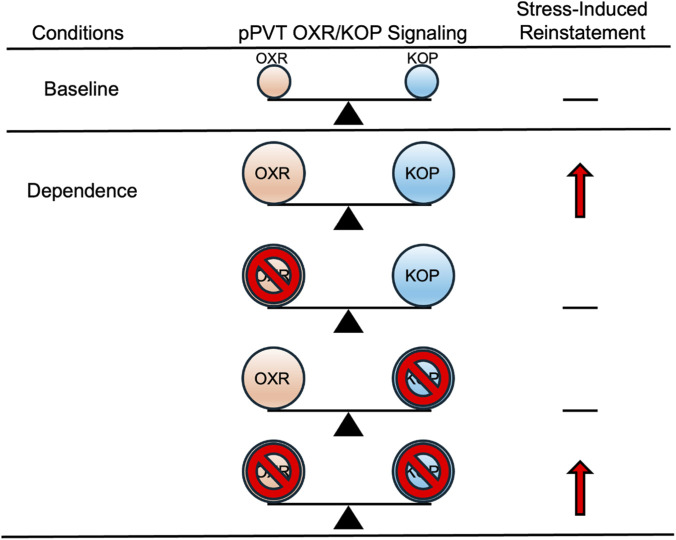
Hypothetical changes in pPVT OXR/KOP balance following dependence. Under normal conditions (i.e., baseline), there is a relative influence of OXR and KOP signaling in the pPVT. Following dependence, the upregulation of OXR/KOP signaling (illustrated by larger circles) drives the stress-induced reinstatement of alcohol-seeking behavior. Individually blocking OXR or KOP signaling decreases the stress-induced reinstatement of alcohol-seeking behavior. The blockade of both OXRs and KOPs reverses this effect, consistent with non-additive and potentially opposing influences of OXR and KOP signaling in the pPVT (Flores-Ramirez et al., 2025).

Taken together, the dense hypothalamic OX/DYN innervation of the pPVT (Chou et al., 2001; Kirouac et al., 2005), its role in stress integration (Hsu et al., 2014; Kirouac, 2021; Penzo and Gao, 2021; Zhou et al., 2013), and the opposing physiological actions of OX and DYN within this region are consistent with a hypothesis in which altered OXR and KOP signaling in the pPVT may contribute to stress-induced alcohol seeking following dependence (Chen et al., 2015; Kolaj et al., 2014; Li and Kirouac, 2008; Matzeu and Martin-Fardon, 2018). Flores-Ramirez et al. (2025) reported that OXR and KOP blockade alone, via intra-pPVT infusions of the DORA TCS1102 and norBNI, dampened stress-induced alcohol reinstatement in dependent rats, while when co-administered their individual effects were modulated (Flores-Ramirez et al., 2025). In addition, the associated increases in *Hcrtr1* and *Oprk1* mRNA that were found in the pPVT are consistent with receptor-level dysregulation of OXR and KOP signaling following chronic alcohol exposure. Figure 2 illustrates these findings by contrasting the individual vs. non-additive effects of combined OXR-KOP blockade in the pPVT on the stress-induced reinstatement of alcohol-seeking behavior following alcohol dependence.

## 10 Multi-target therapy: OXR/KOP blockade as prospective pharmacotherapy for AUD?

Substantial preclinical evidence supports functional roles of OX-DYN in stress and alcohol seeking, and these systems have been identified as pharmacological treatment targets. Recent interest has been seen in repurposing Food and Drug Administration-approved DORAs (e.g., suvorexant, marketed by Merck as Belsomra^®^ for the treatment of insomnia) to treat AUD. OX plays a pivotal role in sleep regulation, and its imbalance in sleep is believed to exacerbate sleep disturbances in AUD (Thakkar et al., 2015; Ziółkowski et al., 2016). Sleep disturbances are reported in individuals with AUD even weeks, months, and years after abstinence. In addition to reducing drug craving via OX1Rs, repurposing DORAs (e.g., suvorexant) may reduce relapse risk by normalizing sleep disturbances via OX2Rs, a direct predictor of alcohol relapse (Crum et al., 2004).

Mechanistically, the chronic elevation of OX levels in abstinent patients has been correlated with alterations of NAc connectivity (Pan et al., 2021). Clinical data show that alcohol-dependent patients exhibited high blood OX concentrations that normalized with sustained abstinence (Ziółkowski et al., 2016). The therapeutic potential of suvorexant and other OXR antagonists is supported by evidence reported in a few translational reviews (Campbell et al., 2020; Khoo and Brown, 2014) and human data that demonstrate clinical benefits of targeting the OX system to reduce alcohol cravings and improve physical and psychological health and sleep quality in AUD and comorbid insomnia (Campbell et al., 2024). Although these are preliminary data from a single case report, it mirrors the promising effects of OXR manipulations on reducing alcohol seeking and reinstatement in preclinical models, including in the pPVT (Flores-Ramirez et al., 2022; Illenberger et al., 2023; Matzeu and Martin-Fardon, 2020a). Early-phase and exploratory clinical trials are currently being conducted to examine the efficacy of suvorexant in normalizing sleep and alcohol craving, underscoring the significance of targeting the OX system for the treatment of AUD (ClinicalTrials.gov: NCT06484075, NCT06326684, NCT03897062, NCT04229095, and NCT06679062).

Whether pharmacotherapies that target both OXRs and KOPs are specific to alcohol remains an important area of research, as OXR signaling also regulates cue-driven seeking for conventional reinforcers, including palatable food, particularly under conditions of high motivational demand or stress (Borgland et al., 2009; Mahler et al., 2014; Mahler et al., 2012). These therapies should selectively diminish alcohol-dependent states while sparing natural reward systems, such as food and water seeking. Indeed, in several paradigms involving high motivation or dependence-like drinking, OXR blockade selectively lowered the motivation to obtain alcohol but not water (Jupp et al., 2011b), sucrose, glucose, saccharin (Brown et al., 2016; Jupp et al., 2011b; Martin-Fardon and Weiss, 2014a; Martin-Fardon and Weiss, 2014b), or bitter-sweet mixtures such as saccharin + quinine (Lei et al., 2016), suggesting that motivation for highly palatable sweet and conventional reinforcers is preserved. OX2R antagonism also selectively decreased alcohol self-administration but not saccharin (Shoblock et al., 2011). Some reports suggest that blocking OX1Rs alone, or combined blockade of OX1Rs and OX2Rs, can reduce both alcohol and non-alcohol reward intake (Anderson et al., 2014).

Interestingly, dual OXR antagonism did not impact palatable food intake in normal baseline states (Khoo et al., 2018). Although it reduced the binge eating of palatable food under conditions of chronic stress and food restriction, it did not reduce standard food pellet intake (Piccoli et al., 2012). This dissociation suggests that OXR signaling might be involved in “reward” (drug or food) intake and seeking under high-demand conditions (Borgland et al., 2009; Mahler et al., 2014), such as dependence-like states or somatic and motivational signs of withdrawal. This supports the view that chronic alcohol consumption reshapes motivational circuitry to bias behavior toward drug rewards over natural rewards (Aston-Jones and Harris, 2004; Kalivas and O'Brien, 2008; Kelley and Berridge, 2002; Wanat et al., 2009) and further indicates that OXR signaling regulates behavior toward highly motivated stimuli, such as alcohol and drugs of abuse (Moorman and Aston-Jones, 2009; Moorman et al., 2017), without broadly suppressing appetitive behaviors.

The clinical application of KOP antagonists in AUD is still in its early stages, but preclinical and human studies suggest that targeting the KOP system may offer a promising therapeutic approach (Dalefield et al., 2022). In humans, the nonselective opioid receptor antagonist naltrexone is currently used as a first-line treatment for AUD. In alcohol-dependent individuals with AUD, positron emission tomography (PET) imaging with a KOP-selective radioligand showed that higher baseline KOP availability was associated with a greater urge to drink and a lower treatment response to naltrexone (De Laat et al., 2019), suggesting that KOP activity may moderate naltrexone’s efficacy. In human participants, Naganawa et al. (2016) examined effects of the KOP antagonist LY2456302 (aticaprant) and used PET scans with the radiotracer 11C-LY2795050 to measure receptor occupancy. The results showed ∼94% and ∼72% KOP occupancy at 2.5 and 24 h post-dose, respectively, confirming the engagement of KOPs and supporting its suitability for further pharmacological targeting (Naganawa et al., 2016). In parallel, Vijay et al. (2018) used PET imaging and found lower KOP availability among an alcohol-dependent cohort relative to controls, suggesting disruptions of DYN circuitry that may contribute to the dysregulation of stress and reward processing in individuals with chronic alcohol use (Vijay et al., 2018). Although leads in humans and animal models position the DYN/KOP system as a promising therapeutic target to reduce alcohol seeking (Mysels, 2009; Reed et al., 2020), an ongoing Yale University-led clinical trial is elucidating how MOP and KOP imbalances may contribute to AUD-related symptoms, such as craving, mood, and withdrawal (ClinicalTrials.gov: NCT05957159). Early evidence underscored the promise of targeting the KOP system to mitigate alcohol-seeking behavior, but further research is needed to establish the safety and efficacy of these compounds in the treatment of AUD. While clinical studies support roles for OX and DYN systems in arousal, stress regulation, and relapse vulnerability, direct evidence linking OX-DYN interactions within the PVT to alcohol relapse in humans remains limited, underscoring the importance of mechanistic preclinical studies.

Although interacting systems that regulate stress and chronic alcohol use are complex, the individual roles of OX and DYN transmission in the pPVT appear to uniquely shape the motivation for alcohol seeking and vulnerability of relapse (Matzeu and Martin-Fardon, 2020a; Moorman and Aston-Jones, 2009). Pharmacological interventions in preclinical and human models suggest these systems may help restore the dysregulation of both the OX/OXR and DYN/KOP systems that is induced by chronic alcohol consumption, and they may mitigate relapse that is triggered by stressful states (Campbell et al., 2024; Flores-Ramirez et al., 2024; Flores-Ramirez et al., 2022). Their interactive contributions in the pPVT were tested post-alcohol dependence (at 3 weeks), indirectly supporting a potential functional interaction among OX and DYN transmission in the pPVT that underlies stress-induced relapse after alcohol dependence and abstinence (Flores-Ramirez et al., 2025). Framing OX transmission as a “driver” of alcohol seeking and DYN as a “break” emphasizes their opposing influences (Mohammadkhani et al., 2024; Muschamp et al., 2014). Fine-tuning these disrupted systems in the pPVT following chronic alcohol use may selectively normalize maladaptive alcohol-seeking behavior (Jupp et al., 2011b; Lopez et al., 2016; Martin-Fardon and Weiss, 2014b; Schank et al., 2012).

## 11 Conclusion

The OX/OXR and DYN/KOP systems are key regulators of stress responsivity, alcohol seeking, and relapse risk (Aston-Jones et al., 2010; Koob, 2015; Moorman et al., 2017). Preclinical and emerging clinical evidence show that OXR and KOP antagonists can reduce alcohol consumption, withdrawal symptoms, and stress-induced reinstatement (Flores-Ramirez et al., 2024; Flores-Ramirez et al., 2022; Kissler and Walker, 2016; Schank et al., 2012). Targeting OXR and KOP signaling may represent a state- and circuit-dependent therapeutic strategy to modulate hyperarousal while countering stress-driven negative affect, namely the “dark side” of addiction (Koob, 2015; Walker and Lawrence, 2016). This approach could be especially effective in remission, when stress and relapse vulnerability are high. Clarifying the roles and dual modulation of OX and DYN in stress-induced alcohol seeking, particularly within pPVT circuits that link stress and reward, is essential for advancing addiction research and guiding targeted pharmacotherapies for AUD.
